# A Critical Appraisal of Growth Hormone Therapy in Growth Hormone Deficiency and Turner Syndrome Patients in Turkey

**DOI:** 10.4274/jcrpe.3209

**Published:** 2016-12-01

**Authors:** Zehra Yavaş Abalı, Feyza Darendeliler, Olcay Neyzi

**Affiliations:** 1 İstanbul University İstanbul Faculty of Medicine, Department of Pediatrics, Division of Pediatric Endocrinology, İstanbul, Turkey; 2 Emeritus Professor

**Keywords:** growth hormone, Growth hormone deficiency, Turner syndrome

## Abstract

Early detection of abnormal growth, identification of the underlying cause, and appropriate treatment of the medical condition is an important issue for children with short stature. Growth hormone (GH) therapy is widely used in GH-deficient children and also in non-GH-deficient short stature cases who have findings conforming to certain indications. Efficacy of GH therapy has been shown in a multitude of short- and long-term studies. Age at onset of GH therapy is the most important factor for a successful treatment outcome. Optimal dosing is also essential. The aim of this review was to focus on challenges in the early diagnosis and appropriate management of short stature due to GH deficiency (GHD) and Turner syndrome. These are the most frequent two indications for GH therapy in Turkey approved by the Ministry of Health for coverage by the national insurance system.

## INTRODUCTION

Today, Turkey’s population exceeds 78 million with children and adolescents constituting nearly one third of the total population (1). Being the best indicator of general health and well-being, appropriate monitoring of growth status and thus early identification of abnormal growth seems fundamental to health care in such a youthful population given the likelihood of management of underlying medical conditions, optimizing attainment of good health and normal adult height (2).

Accurate assessment and monitoring of growth in children on the basis of length or height according to age, weight, body mass index (BMI), and height velocity (HV) with respect to reference populations is of critical importance for early identification and proper evaluation of remediable conditions associated with a reduced growth rate and/or short stature. Early initiation of treatment in children with these conditions would possibly enable them to achieve their potential to reach an adult height within the normal population range (2,3).

The further below -2.0 standard deviations [SD] (2.5 percentile) an individual’s growth falls, the more likely it is that there is an underlying pathological condition associated with short stature. The possibility to reach the genetically determined height potential also becomes more limited in such individuals (2,4).

According to data from the Turkish Demographic and Health Survey, an improvement was noted in the nutritional status of children over the years with a dramatic decrease in the frequency of undernutrition- and malnutrition-related growth failure (5). Additionally, the recent report of the Turkey Childhood Obesity Surveillance Initiative in 2013 stated that the frequency of children (aged 7-8 years) with a height standard deviation score (SDS) below -3 SD is 0.1% and that of those with a height below -2 SD is 2.3% (6). Nonetheless, despite this decline in the frequency of short children over the years, there are still several underlying causes of short stature other than those related to nutrition that need to be evaluated by pediatric endocrinologists.

Pituitary-derived human GH was first used as replacement therapy in a child with hypopituitarism (7). In subsequent years, with the development of the recombinant human GH (rhGH) and its introduction to clinical use, there has been a marked increase in the scope of GH treatment (8). Indications for GH therapy extended from replacement therapy in GH deficiency (GHD) to many diseases in which short stature is not secondary to GHD (9). Among several indications of GH treatment (10,11), GHD and Turner syndrome (TS) are the two most frequent conditions approved for GH treatment by the Turkish Ministry of Health. In this context, it must also be noted that under the Turkish national health system, all individuals are covered for their health expenditures, including GH treatment.

In Turkey, there are 73 pediatric endocrinology centers with a total number of 200 endocrinologists (according to 2015 data) (12). The Turkish Pediatric Endocrinology and Diabetes Society (TPEDS) is an active society that holds regular annual scientific meetings and that also organizes periodic educational conferences on growth disorders among other endocrine issues. TPEDS also has many publications including consensus reports and expert reports relating to the diagnosis and treatment of GH deficiency (13,14,15,16,17,18,19,20). Although there are many studies on growth disorders, early diagnosis of the underlying cause of short stature and especially the diagnosis of conditions that may benefit from GH treatment is still a challenge. The aim of this review is to focus on the early diagnosis and appropriate management of GHD and TS patients, with emphasis on optimum GH treatment, compliance to therapy, and transition to adult healthcare.

### Growth Hormone Therapy in Children with Growth Hormone Deficiency

The diagnosis of GHD is usually based on the combination of auxological findings and poor growth velocity, confirmed by a low insulin-like growth factor-1 (IGF-1) concentration and the results of GH provocative testing using arginine, clonidine, glucagon, insulin, or L-dopa, with a peak GH cut-off set at 10 μg/L in at least two tests (21). In recent reports, it has been recommended that a cut-off peak level of 7 μg/L would be appropriate using the recent monoclonal GH antibodies (22).

Age at initiation of GH treatment has been shown to negatively correlate to response to therapy emphasizing the need of early diagnosis and treatment (23). In a multicenter study using the Turkish data in the KIGS database, 1008 cases with GHD were evaluated demographically and by treatment results of the first year. It was concluded that the diagnosis of GHD was late in these cases. Mean age (minimum-maximum) at onset of therapy was 11.3 (5.4-15.1) years in idiopathic GHD cases. Mean height SDS was -3.1 (-5.2 to -1.9) in these patients (24). Mean GH dose was 28 (21-34) µg/kg/d. The response expressed as delta height SDS was 0.6/1 year and 1.1/3 years of therapy which was within expected limits but at the lower end (25). To standardize the diagnosis and treatment of GHD in Turkey, TPEDS is working on new consensus guidelines for diagnostic procedures and treatment of Turkish children with GHD (26,27).

Several prevailing guidelines for the diagnosis and treatment of GHD in children have been published (23,28,29,30,31,32). However, differences in diagnostic procedures and treatment strategies among countries and even among centers in the same country continue to exist (33,34). The European Society of Pediatric Endocrinology (ESPE) recommends 25-35 µg/kg/d as starting doses of rhGH in GHD, while the dose recommended by the American Society of Pediatric Endocrinology is 43 µg/kg/d (32). In a recent study on current practice in diagnosis and treatment of GHD in childhood in Turkey, the most frequently used starting dose of rhGH was reported to be 25-30 µg/kg/d in prepubertal children and 30-35 µg/kg/d in pubertal children, consistent with ESPE recommendations (35).

In this survey, rhGH dose adjustment was primarily based on growth velocity as recommended by consensus publications with monitoring response and change in HV every 3-6 months. Cessation of rhGH therapy was primarily done according to HV and bone age advancement (35). There are different practices in European countries as well despite the published guidelines. In an audit from European countries, the range of starting doses of GH in GHD patients was broad (11-50 µg/kg/d) and approximately 60% of the units from EU cenetrs preferred the dose of 30 µg/kg/d (36). Recent multicenter Italian study reported that median dose of GH was 33 µg/kg/d in GHD patients (37).

### Growth Hormone Therapy in Children with Turner Syndrome

TS, the most common sex chromosome abnormality in females with an estimated prevalence of 1/2500 occurs as a result of partial or complete absence of one X chromosome, leading to a combination of characteristic phenotypic features including short stature (3).

The average adult height deficit in untreated women with TS is 20-21 cm compared to normal adults, with an average height of 147 cm (38). The therapeutical GH doses exceeding the physiological dose have been shown to improve growth velocity (39). However, the effect of GH therapy on the final height is substantially variable depending on several clinical and genetic factors such as polymorphisms related with GH receptor and/or IGFBP3 gene, young age, and bone age delay at the start of GH treatment. GH dose, duration of GH treatment, maternal X chromosome origin, target height, and good first-year height response to GH treatment, use of oxandralone and weekly number of injections have also been shown to affect treatment results (40,41,42). Since the growth response is known to decrease over the years of GH treatment, not only the good first-year response to GH therapy but also maintenance of the good response has been considered necessary to be able to achieve final height. Hence, increments in GH dose in patients at risk of poor GH response have been considered to be effective in terms of cost and safety via generating better short- and long-term growth response (40,43).

Prompt initiation of GH treatment has been recommended in TS patients as soon as growth failure is demonstrated, even in infancy. Initiation of therapy at a young age, optimally while the child is still within normal length/height values for age, has been reported to improve the outcome, to decrease overall cost, and also to yield higher psychosocial benefits such as being closer to peers in height throughout life. With early treatment, puberty can also be more likely to be initiated at a normal age and GH therapy is likely to be terminated earlier (41).

Hence, early initiation of GH treatment (at a dose of 50 μg/kg/d) and induction of puberty at a normal physiological age were emphasized as important to achieve a taller adult stature (3). Younger age and tall height at GH therapy onset, tall parental heights, better first-year responsiveness to GH, long duration of therapy, and a high GH dose were reported amongst the factors predictive of taller adult height (44).

Data from a past study in Turkish TS patients revealed final height of non-GH treated TS cases to be 141.6 cm, which was 18.4 cm lower than average final height of women without TS (38).

A national survey with a multicenter design conducted in 2003 revealed that only 32% out of 367 TS patients received GH therapy. Of these, regular GH therapy was given to 72%. Advanced chronological age and/or bone age (35%) and lack of insurance benefits (61%) were the two most important factors in not initiating GH therapy (20).

Evaluation of the data of 70 TS patients registered from 11 centers in Turkey in the KIGS database who received GH in a dose of 33 (0.23/46) µg/kg/d subcutaneously, 6-7 times per week, with onset of therapy at age 12.5 (7.1/15.6) years revealed a non-significant increase in HV [6.3 (4.3/8.5) cm/year in the first year and 5.9 (3.6/8.7) cm/year in the second year]. Height SDS improved to -3.4 (-4.6/-2.2) in the first year and to -2.7 (-4.2/-1.6) in the 2nd year of therapy in the longitudinally followed TS patients (45). Thus, in Turkish children, there was a delay in both age at diagnosis of TS and age of onset of GH therapy as compared to other reports. The dose of GH used and the response were also lower (44). In an Italian multicenter study, median GH dose used for TS was 43 µg/kg/d (37). In another recent multicenter study, age at onset of GH therapy was 9.4±2.6 years, the dose of GH used was 50 µg/kg/d, and delta height SDS over 1 year was 0.4 SD (46). In a very recent study from Turkey, evaluation of 842 patients with TS from 35 centers revealed that mean age at diagnosis of TS was 10.5±4.8 years with initiation of rhGH therapy shortly after diagnosis, at 10.7±3.5 years (47). The number of GH treated patients was 615 in this cohort. The age at initiation of GH therapy in girls with TS improved in Turkey by years. We think that it is related to increased number of pediatric endocrinologist in Turkey and more frequent referral of patients with short stature to pediatric endocrinologists. The other important factor is continuous post-graduate education to pediatricians, family doctors, and pediatric endocrinologists. Last but not the least, almost all children under 18 years have insurance.

Evaluation of GH treatment response in TS patients either according to prediction models or HV target charts (48,49) will enable the clinician to evaluate the confounding factors and use optimum dose adjustment and also to be able to provide realistic information to the child and the parents.

### Adherence to Growth Hormone Therapy

Long-term GH replacement therapy in GHD, starting at the time of diagnosis, typically from childhood throughout adolescence and into adulthood is recommended by the GH Research Society and the European Society of Pediatric Endocrinology (23,50). Accordingly, as in non–life-threatening chronic conditions, problems with adherence to GH therapy may be exacerbated due to long treatment duration along with factors specific to GH therapy such as the need for subcutaneous injections on a daily basis, inadequate training in device technique, as well as lack of immediate therapeutic benefits (50,51,52,53).

Data suggest that poor adherence is frequent among patients receiving GH therapy, despite the fact that continuous, long-term adherence is essential to achieve optimal therapeutic results with GH (54,55). Given the association of injection frequency with growth response, lower adherence to GH therapy has been associated with significantly reduced height velocities (53,55).

**Figure 1:**
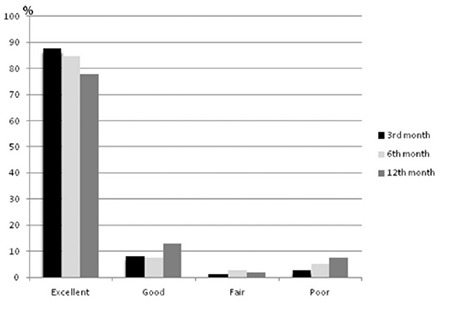
Percentage of adherence and decrement in adherence in the first year of growth hormone therapy (51) Adherence segments based on percentage of doses omitted at each evaluation period: Excellent: 0%, good: 5%, fair: 5 to 10%, and poor: >10% Adapted from Aydın BK, Aycan Z, Sıklar Z, Berberoğlu M, Ocal G, Cetinkaya S, Baş VN, Kendirci HN, Cetinkaya E, Darcan S, Gökşen D, Evliyaoğlu O, Sükür M, Baş F, Darendeliler F. Adherence to growth hormone therapy: results of a multicenter study. Endocr Pract 2014;20:46-51.

Data of 217 GH-naïve patients from 6 pediatric endocrinology clinics in Turkey revealed a decrement in adherence to GH therapy during a 12-month period (Figure 1). Excellent adherence ratio in the first 3 months was 88%, however it was 78% at the end of the first year. The poor adherence ratio was increased 3% to 7.5% in one-year period of treatment. In this study, patients with excellent and good adherence had better response to GH therapy. Also, growth velocity SDS was shown to correlate negatively with number of missed injections and positively with delta IGF-1 levels (51).

Hence, in patients who do not adhere to the prescribed GH therapy, there is a risk that they will not achieve the physical and psychological benefits of treatment and it is therefore important to consider non-adherence to contribute to variability in response to GH therapy and to be a possible cause in all cases of treatment failure.

In conclusion, we believe it is fair to state that the continuous education programs for medical doctors and other health workers conducted by the TPEDS, in addition to the several initiative efforts of our pediatric endocrinologist colleagues in Turkey towards improving the state regulations, have contributed much to the improvement and expansion of GH treatment in GHD and TS in Turkish children. However, we are still faced with challenges and barriers against early diagnosis and optimal dosing in Turkey. Continuous growth monitoring and correct evaluation of short stature or poor growth and referral to secondary or tertiary centers is mandatory for early diagnosis of the underlying etiology of short stature. Once indication of GH therapy is made, correct dosing and correct interpretation of growth velocity is important for optimum outcome.

## Acknowledgments

The authors would like to thank to Prof. Şule Oktay, MD, PhD and Çağla Ayhan, MD from KAPPA Consultancy Training and Research Ltd. for their medical writing services funded by Novo-Nordisk Turkey.

## Ethics

Peer-review: Internally peer-reviewed.
